# CpG-C ODN M362 as an immunoadjuvant for HBV therapeutic vaccine reverses the systemic tolerance against HBV

**DOI:** 10.7150/ijbs.62424

**Published:** 2022-01-01

**Authors:** Huajun Zhao, Qiuju Han, Ailu Yang, Yucan Wang, Guan Wang, Ang Lin, Xiao Wang, Chunlai Yin, Jian Zhang

**Affiliations:** Institute of Immunopharmaceutical Sciences, School of Pharmaceutical Sciences, Shandong University, Jinan, China.

**Keywords:** chronic hepatitis B, CpG-C ODN, therapeutic vaccine, adjuvant, immune-tolerance, CXCR5^+^ CD8^+^ T cells

## Abstract

Chronic Hepatitis B virus (CHB) infection is a global public health problem. Oligodeoxynucleotides (ODNs) containing class C unmethylated cytosine-guanine dinucleotide (CpG-C) motifs may provide potential adjuvants for the immunotherapeutic strategy against CHB, since CpG-C ODNs stimulate both B cell and dendritic cell (DC) activation. However, the efficacy of CpG-C ODN as an anti-HBV vaccine adjuvant remains unclear. In this study, we demonstrated that CpG M362 (CpG-C ODN) as an adjuvant in anti-HBV vaccine (cHBV-vaccine) successfully and safely eliminated the virus in HBV-carrier mice. The cHBV-vaccine enhanced DC maturation both *in vivo* and *in vitro*, overcame immune tolerance, and recovered exhausted T cells in HBV-carrier mice. Furthermore, the cHBV-vaccine elicited robust hepatic HBV-specific CD8^+^ and CD4^+^ T cell responses, with increased cellular proliferation and IFN-γ secretion. Additionally, the cHBV-vaccine invoked a long-lasting follicular CXCR5^+^ CD8^+^ T cell response following HBV re-challenge. Taken together, CpG M362 in combination with rHBVvac cleared persistent HBV and achieved long-term virological control, making it a promising candidate for treating CHB.

## Introduction

Chronic Hepatitis B virus (CHB) infection currently affects approximately 240 million people worldwide 1. Patients with CHB are at higher risk for developing cirrhosis and hepatocellular carcinoma (HCC) 2. Current antiviral therapies, such as pegylated interferon alpha2a (PegIFN) and nucleoside/nucleotide analogues (NAs), are unable to achieve efficient hepatitis B surface antigen (HBsAg) loss 3. Therefore, a novel strategy involving immunomodulation needs to be developed to achieve long-term virological control in CHB.

The pathological basis of CHB infection is the persistent presence of HBV covalently closed circular DNA (cccDNA) in the nuclei of infected hepatocytes, as well as an immunosuppressive environment in the infected liver 4, 5. Both the innate and adaptive immune responses are involved in the pathogenesis of CHB infection, and influence its clinical outcome 5-7. However, HBV-specific immune responses are characteristically weak, transient and nearly undetectable in CHB patients. Furthermore, neither the prophylactic HBV vaccine alone nor its combination with other antiviral compounds has successfully eliminated HBV-infected cells in CHB. Since the ideal HBV therapy should reverse host immune tolerance to the virus and recover the function of HBV-specific T cells, an effective adjuvant may improve the success of therapeutic HBV vaccines.

CpG oligodeoxynucleotide (CpG ODN) are synthetic single-stranded DNA molecules containing unmethylated cytosine-guanine dinucleotide (CpG) motifs. As an agonist for Toll-like receptor 9 (TLR9), immuno-stimulatory CpG activates antigen-presenting cells (APCs) to produce inflammatory cytokines, thus inducing the T helper type 1 (Th1)-type immune response via TLR9 activation 8-10. At least three major classes of CpG-ODNs have been characterized according to their backbone, sequence, and immuno-stimulatory properties: class A (D-type), class B (K-type), and class C 11. CpG-A ODNs such as CpG 1585, CpG 2216, and CpG 2336 activate plasmacytoid dendritic cells (pDCs) to produce interferon-α (IFN-α), but fail to induce B cell activation 8, 9. In contrast, CpG-B ODNs such as CpG 1826, CpG 2006, and CpG 7909 strongly induce B cells to produce interleukin-6 (IL-6), but promote pDC maturation with absence of IFN-α secretion 8, 12. Finally, CpG-C ODNs combine the features of classes A and B, activating both pDCs and B cells 13. CpG ODNs have been widely used as adjuvants for various antiviral vaccines against hepatitis C virus (HCV), human immunodeficiency virus (HIV), and HBV, as well as cancer cells 14-16. The prophylactic HBV vaccine Engerix-B combined with CpG 7909 (CpG-B ODN) resulted in higher HBs antibody (anti-HBs) titers and enhanced affinity maturation to improve the avidity of anti-HBs 16. In addition, a two-dose schedule of HEPLISAV-B, comprised of recombinant HBsAg and 1018 ISS (CpG-B ODN), demonstrated a significantly higher rate of protection (95%) compared to that observed with Engerix-B (81%) in a phase III trial 17,18. Systemic administration of CpG 1826 (CpG-B ODN) inhibited HBV replication by inducing type I IFNs in HBV transgenic mice 19, 20. In another study, administration of nanoparticles containing unmethylated CpG-A ODNs (HBV-CpG) exerted a strong immuno-stimulatory effect on DCs, NK cells, and T cells *in vivo*, and led to viral clearance in HBV-carrier mice 21. Taken together, these studies indicate that CpG-A and -B agonists may function as potent immunomodulatory agents against CHB infection by augmenting HBV-specific T or B cell responses. In a phase 1b multicenter trial, CpG 10101 (CpG-C ODN) activated the immune response along with secretion of IFN-α to reduce HCV RNA levels in a dose-dependent manner 22. Furthermore, intra-tumoral SD-101 (CpG-C ODN) administration in combination with low-dose radiation in a phase 1/2 trial promoted the generation of tumor-specific CD8^+^ and CD4^+^ effector T-cells, and reduced the abundance of T regulatory cells (Tregs) in the tumor microenvironment, which in turn led to complete tumor regression in both treated and untreated tumor sites 23. However, the therapeutic utility of CpG-C ODNs as HBV vaccine adjuvants remains unclear.

Exhausted CD8^+^ T cells play a critical role in the development of CHB infection. Recent studies have reported that CXCR5-expressing CD8^+^ T cells are partially exhausted with strong antiviral activity 24-27, producing higher levels of IFN-γ, TNF-α, IL-21, and granzymes during lymphocytic choriomeningitis virus (LCMV), HIV, and other chronic infections than CXCR5^-^ CD8^+^ T cells. Additionally, CXCR5^+^ CD8^+^ T cells can migrate into B cell follicles, thereby supporting B cell activation, affinity maturation, and antibody production 26-28. CXC chemokine ligand 13 (CXCL13) exclusively binds to chemokine receptor CXCR5 expressed on CD8^+^ T cells to help recruit CXCR5^+^ T cells to the inflammatory site, thus coordinating both humoral and cellular immune responses 29,30. Moreover, Li *et al.* reported that elevated expression of CXCL13 facilitated the recruitment of CXCR5^+^ CD8^+^ T cells in the liver, which in turn inhibited HBV replication and regulated production of B cell antibodies in patients with CHB 27. However, whether CpG-C ODNs can sustain HBV control by inducing the follicular CXCR5^+^ CD8^+^ T cell response is unknown.

Previous studies have demonstrated that an HBV-carrier mouse model of persistent HBV infection can be generated via injection of an AAV-HBV vector 31-34. These mice do not mount the specific immune response to conventional HBV vaccines, thus mimicking the immune tolerance exhibited in human CHB 32, 33, 35. Therefore, the goal of the current study was to evaluate the therapeutic feasibility of using CpG-C ODN M362 as an HBV vaccine adjuvant using AAV/HBV-transduced HBV-carrier mice.

## Materials and Methods

### Animals and reagents

Five- to six-week-old male C57BL/6J mice were purchased from the Beijing HFK Bioscience Co. Ltd (Beijing, China). All animals were treated in accordance with the Guidelines for the Care and Use of Laboratory Animals of the Ethical Committee of Shandong University and the protocol was approved by the Institutional Animal Care and Use Committee of Shandong University. rHBVvac (Hansenula polymorpha) was purchased from Dalian Hissen Bio-pharm. Co., Ltd. (Dalian, China) and CpG M362 was obtained from Invivogen (San Diego, CA, USA).

### HBV-carrier mouse model

HBV-carrier mice were generated by AAV-HBV transduction using pAAV/HBV1.2 plasmids carrying full-length HBV DNA (kindly provided by Pei-Jer Chen, National Taiwan University, Taiwan), as previously described 22,32,35,36. Serum HBsAg levels were measured 6 weeks after hydrodynamic injection of pAAV/HBV1.2 plasmids, and HBV-carrier mice were identified as those with serum HBsAg levels > 500 ng/mL (1000 ng/mL = 1.14 IU/mL).

### Vaccination and HBV re-challenge

**Figure 1:**
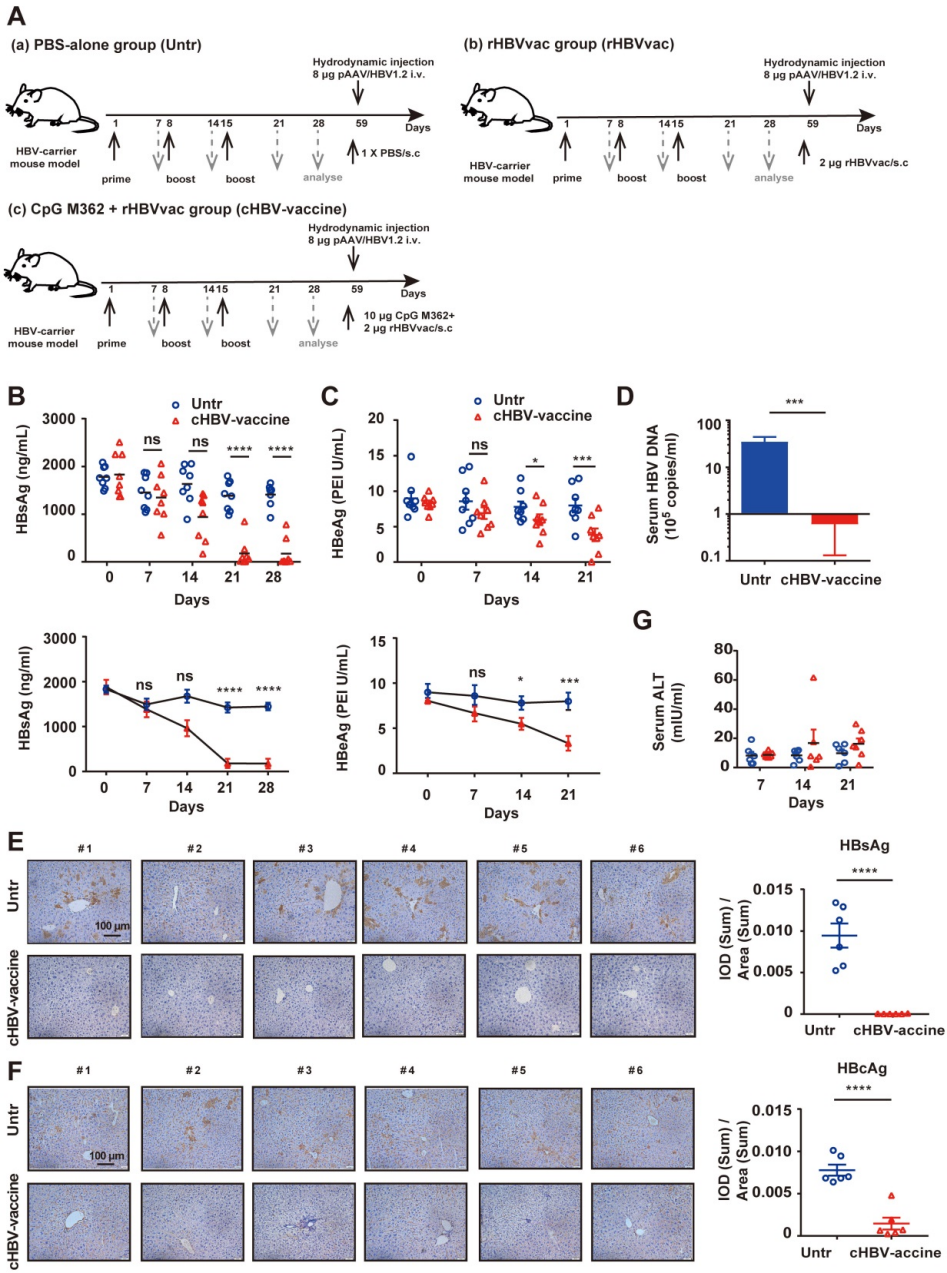
** Immunization with cHBV-vaccine efficiently eliminated HBV in the carrier mice.** Schematic illustration of HBV vaccination protocol in HBV-carrier mice. Mice were immunized subcutaneously with PBS (Untr), 2 μg rHBV vaccine (rHBVvac), or 2 μg rHBVvac combined with 10 μg CpG M362 (cHBV-vaccine) weekly for 3 weeks (on days 1, 8, and 15). Blood samples were drawn weekly from the lateral tail vein. For the long-term memory immune response assay, HBV-carrier mice were re-challenged with hydrodynamic injections of 8 μg pAAV/HBV1.2 plasmid on day 59 after the first vaccination. (B) Post-immunization serum levels of HBsAg, measured by ELISA and compared with two-way ANOVA. (C) Post-immunization serum levels of HBeAg, measured by ELISA and compared with two-way ANOVA. (D) Number of HBV DNA copies on day 21 post-immunization. (E) HBsAg expression in hepatocytes on day 21 post-immunization via IHC staining (200 × magnification). (F) HBcAg expression in hepatocytes on day 21 post-immunization via IHC staining (200 × magnification). (G) Serum levels of ALT monitored on days 7, 14, and 21 post-immunization. (Normal serum ALT levels are < 40 mIU/mL). All data are expressed as mean ± SEM of biological replicates. * p < 0.05, ** p < 0.01, *** p < 0.001, **** p < 0.0001 versus untreated mice.

As shown in Fig. 1A, HBV-carrier mice were immunized subcutaneously with phosphate-buffered saline (PBS) (Untr), 2 μg rHBV vaccine (rHBVvac), or 2 μg rHBVvac combined with 10 μg CpG M362 (cHBV-vaccine) weekly for 3 weeks (on days 1, 8, and 15). Blood samples were drawn weekly from the lateral tail vein, and the serum was stored at -80 ºC until further analysis. For the long-term memory immune response assay, HBV-carrier mice were re-challenged with hydrodynamic injections of 8 μg pAAV/HBV1.2 plasmid on day 59 after the first vaccination.

### Generation and stimulation of bone marrow-derived dendritic cells (BMDCs) *in vitro*

Murine BMDCs were generated as previously described 37, and CD11c^+^ BMDCs were identified and enriched using fluorescence-activated cell sorting (FACS) (> 90%). To assess their antigen-presenting ability, the isolated BMDCs were incubated with CpG M362 for 12 h, and surface expression of CD86 and MHC-II was analyzed by flow cytometry.

### Chemiluminescent immunoassay (CLIA) and Enzyme-linked immunosorbent assay (ELISA)

Specific ELISA kits for serum HBsAg (Autobio, Zhengzhou, China), HBeAg (Autobio), anti-HB (Wantai Biological Pharmacy Enterprise Co., Ltd., Beijing, China), and TGF-β1 (Multi Sciences Biotech Co., Ltd., Hangzhou, China) were used according to the manufacturers' instructions. Serum alanine aminotransferase (ALT) levels were determined using a commercially available assay kit (Nanjing Jiancheng Bioengineering Institute, Nanjing, China).

### HBV DNA detection

HBV DNA was extracted from 50 μL mouse serum using the HBV DNA quantitation kit according to the manufacturer's instructions (Daan Gene, Guangzhou, China) and measured by quantitative PCR using UltraSYBR Mixture (CW Biotech, Beijing, China) with a Lightcycler® 96 (Roche, Basel, Switzerland).

### Immunohistochemistry (IHC)

Intrahepatic HBcAg and HBsAg were detected in mouse liver sections by immunostaining with anti-HBcAg mAb (#GB058604, Gene Tech Co., Ltd., Shanghai, China) and anti-HBs mAb (#GB058604, Gene Tech Co., Ltd.), respectively. Horseradish peroxidase-conjugated goat anti-mouse IgG (#ZB-2305, ZSGB-bio Co. Ltd, Beijing, China) was used according to the manufacturer's instructions. HBcAg^+^ and HBsAg^+^ hepatocytes were counted using Image-Pro Plus software (Media Cybernetics, Rockville, MD, USA).

### Cell isolation

Single-cell suspensions from the liver, spleen, and draining lymph nodes (dLNs) were isolated as previously described 36. Briefly, the PBS-perfused liver was passed through a 200-μm nylon cell strainer to obtain the single-cell suspension, which was centrifuged at 100 × rcf for 1 min to remove hepatocytes. Then, the supernatant was centrifuged at 400 × rcf for 10 min to collect residual cells, which were layered over 40% Percoll (GE Healthcare, Uppsala, Sweden). Hepatic mononuclear cells (MNCs) were harvested after centrifugation at 400 × rcf for 10 min, followed by red blood cell (RBC) lysis and washing. The spleens and dLNs were passed through a 200-μm nylon cell strainer, single cells were harvested, followed by RBC lysis, and washing.

### Flow cytometry

Single-cell suspensions were pre-incubated with Fc-receptor blocking solution (anti-mouse CD16/32, **#**14-0161-82, eBioscience, California, USA) for 30 min, and stained with the fluorochrome-conjugated antibodies conjugates at 4˚C for at least 1h. The following mAb were used: FITC-anti-CD4 (**#**4313007), FITC-anti-CD8α (**#**4271604), FITC-anti-CD11c (**#**E00155-1631), PE-anti-CD69 (**#**E01333-1634), PE-anti-CD8α (**#**E01038-1633), PE-anti-CD86 (**#**E01369-1634), PE-anti-CD107a (**#** E01430-1632), PE-anti-B7H1 (**#**4276913), PE-anti-CD49d (**#**E01278-1635), PE-anti-CXCR5 (**#**E16203-104), Percp-Cy5.5-anti-CD3e (**#**4304569), Percp-Cy5.5-anti-FOXP3 (**#**E08398-1634), APC-anti-CD25 (**#**E07106-1634), APC-anti-CD80 (**#**4329685), APC-anti-PD-1 (**#**4344425), eFluor 450-anti-Ki-67 (**#**1928649), eFluor 450-mouse hematopoietic lineage (**#**2324728) from eBioscience (California, USA); PE-anti-MHC-II (**#**107608), Percp-Cy5.5-anti-CD11a (**#**101124), BV421-anti-LAG3 (**#**125221), PE-Cy7-anti-TIGIT (**#**142107), AF700-anti-mouse CD317 (**#**127037), APC-Cy7-anti-CD107a (**#**121615) from Biolegend (San Diego, USA); PE-CF594-anti-CD8α (**#**562283), BV510-anti-CD11a (**#**563669) from BD Bioscience (Bedford, USA). All data were acquired on BD FACS Calibur, BD FACS Celesta or BD FACS Aria III flow cytometer and analyzed with FlowJo software (BD Life Sciences, Franklin Lakes, NJ, USA).

### Statistical analysis

Data were analyzed using GraphPad Prism version 6 software (GraphPad Software, La Jolla, CA, USA) and groups were compared using unpaired two-tailed t-tests and two-way analysis of variance (ANOVA). A p-value < 0.05 was considered statistically significant (*p < 0.05, **p < 0.01, ***p < 0.001, ****p < 0.0001).

## Results

### cHBV-vaccine efficiently eliminated HBV in carrier mice

HBV-carrier mice were used to evaluate the efficiency and safety of the cHBV-vaccine (Fig. 1A). The results confirmed that rHBVvac alone did not eliminate HBV (Fig. S1A-C). Compared to serum HBsAg and HBeAg levels in untreated mice, those in cHBV-vaccinated mice decreased significantly and remained at low levels after the third immunization (Fig. 1B, 1C). Furthermore, serum HBV DNA, and intrahepatic HBsAg and HBcAg were nearly undetectable in cHBV-vaccinated mice (Fig. 1D-F). However, none of these treatments induced the generation of anti-HBs (Fig. S1C). In addition, the serum ALT remained at baseline levels, indicating that cHBV vaccination did not result in liver injury (Fig. 1G). Taken together, these results indicated that CpG M362 is a promising and safe adjuvant for use in HBV therapeutic vaccines.

### CpG M362 promoted the maturation and antigen-presenting ability of DCs

**Figure 2:**
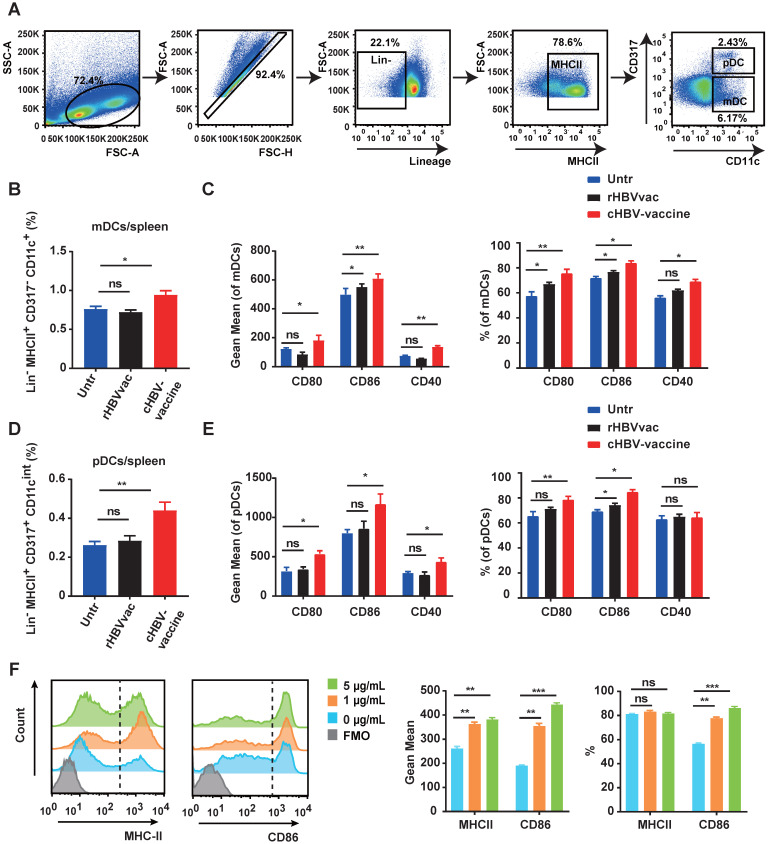
** CpG M362 promoted the maturation and antigen presenting ability of DCs.** HBV-carrier mice were immunized subcutaneously with PBS (Untr) and 2 μg rHBVvac combined 10 μg CpG M362 (cHBV-vaccine) weekly for 3 weeks, separately. (A) The gating strategy of mDCs and pDCs. (B) Proportion of splenic mDCs (Lin^-/-^ MHCII^+^ CD317^-^ CD11c^+^) on day 21 post-immunization. (C) Flow cytometry results showing expression of CD80, CD86, and CD40 on splenic mDCs. (D) Proportion of splenic pDCs (Lin^-/-^ MHCII^+^ CD317^+^ CD11c^int^) on day 21 post-immunization. (E) Flow cytometry results showing expression of CD80, CD86, and CD40 on splenic pDCs. (F) MHC-II and CD86 expression on BMDCs incubated with different doses of CpG M362 for 12 h. All data are expressed as mean ± SEM (n ≥ 8). * p < 0.05, ** p < 0.01 versus untreated mice.

As professional APCs, DCs play a vital role in generating antigen-specific T-cell responses against chronic HBV infection 38. As shown in Fig. 2A-C, the spleen of cHBV-vaccinated mice exhibited higher proportions of myeloid DCs (mDCs) (Lin^-/-^ MHCII^+^ CD317^-^ CD11c^+^) and pDCs (Lin^-/-^ MHCII^+^ CD317^+^ CD11c^int^) compared to those in untreated mice. Moreover, the expression of co-stimulatory molecules CD80, CD86, and CD40 was upregulated on mDCs and pDCs in spleen following treatment with the cHBV-vaccine compared to that in untreated mice (Fig. 2D, 2E). A similar phenomenon was observed on mDCs and pDCs in dLNs (Fig. S2A-D). However, treatment with rHBVvac alone did not efficiently enhance DC activation (Fig. 2B-E, Fig. S2A-D). To further confirm the stimulatory effect of CpG M362 on DCs, BMDCs were generated* in vitro* and treated with different doses of CpG M362. Consistently, CpG M362 increased the surface expression of MHC-II and CD86 on BMDCs compared to that in the unstimulated controls (Fig. 2F). These results confirmed that the CpG M362 adjuvant promoted the maturation and antigen-presenting ability of DCs *in vivo* and *in vitro*.

### cHBV-vaccine amplified robust Ag-specific CD8^+^ and CD4^+^ T cell responses

**Figure 3:**
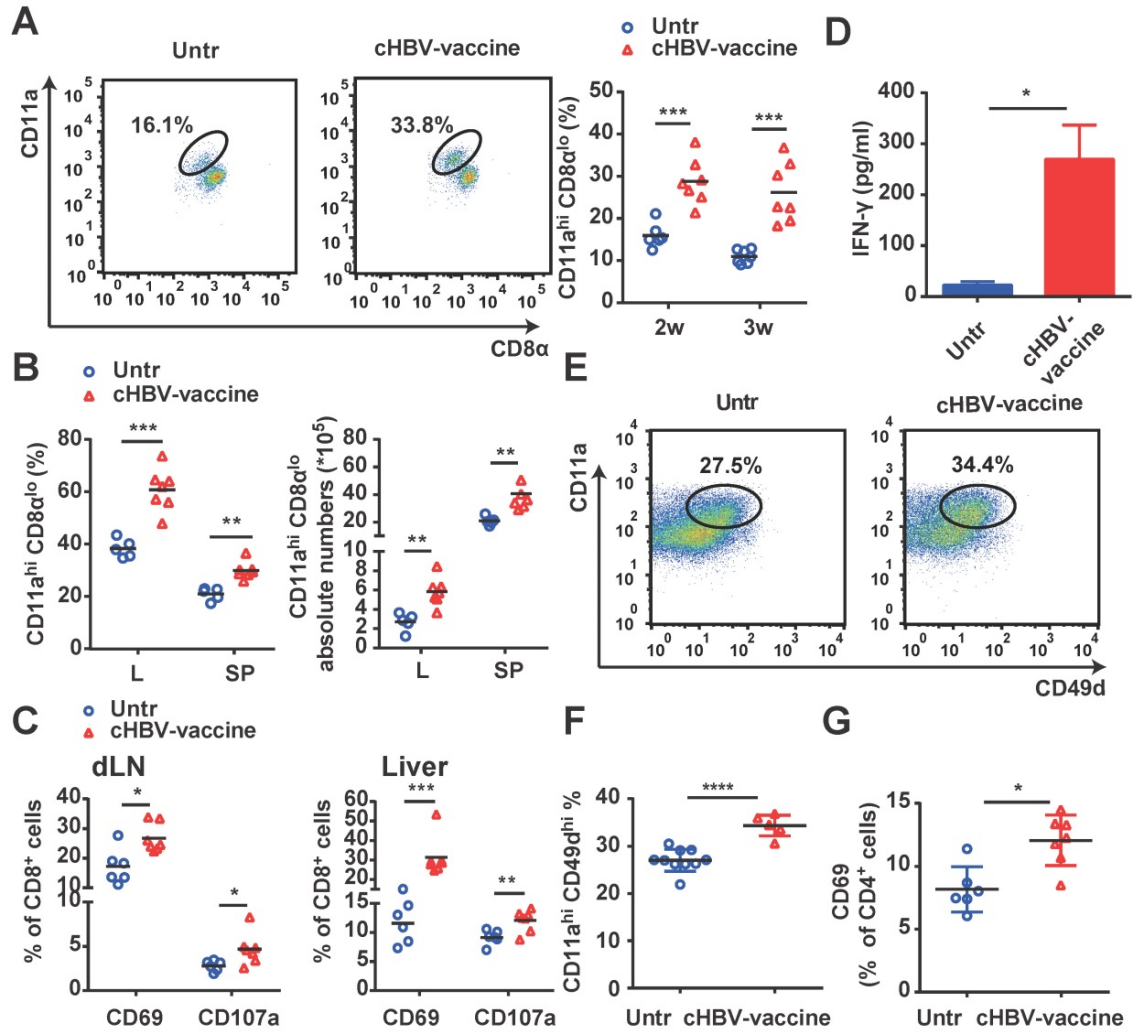
** cHBV-vaccine amplified HBV-specific CD8^+^ T and CD4^+^ T cell responses.** HBV-carrier mice were immunized subcutaneously with PBS (Untr) and 2 μg rHBVvac combined with 10 μg CpG M362 (cHBV-vaccine) weekly for 3 weeks, separately. (A) The proportion of CD11a^hi^ CD8α^lo^ cells among CD8^+^ T cells from peripheral blood. (B) The proportion and abundance of CD11a^hi^ CD8α^lo^ cells among CD8^+^ T cells from the liver and spleen on day 21 post-immunization. (C) The frequency of CD8^+^ CD69^+^ T cells and CD8^+^ CD107a^+^ T cells in dLNs (left) and liver (right) on day 21 post-immunization. (D) Serum levels of IFN-γ monitored by ELISA on day 21 post-immunization. (E-G) The proportion of CD4^+^ CD11a^hi^ CD49d^hi^ T cells (E, F) and CD4^+^ CD69^+^ T cells (G) in the spleen on day 21 post-immunization. All data are expressed as mean ± SEM (n ≥ 5). * p < 0.05, ** p < 0.01, *** p < 0.001, **** p < 0.0001 versus untreated mice.

Since HBV-specific CD8^+^ and CD4^+^ T cells play a crucial role in controlling HBV progression 35, 39, we evaluated whether vaccination enhanced the magnitude and quality of HBV-specific CD8^+^ and CD4^+^ T cell responses. As shown in Fig. 3A and 3B, cHBV-vaccinated mice exhibited a significantly higher proportion of HBV-specific CD11a^hi^ CD8α^lo^ cells 35, 36, 40 in their peripheral blood, liver, and spleen, compared to untreated mice. Furthermore, cHBV-vaccination resulted in a marked increase in the expression of CD69 and CD107a on CD8^+^ T cells (Fig. 3C), along with increased IFN-γ secretion (Fig. 3D). In addition, the abundance of HBV-specific CD4^+^ CD11a^hi^ CD49d^hi^ CD4^+^ T cells 35, 41 was also significantly higher in the spleens of cHBV-vaccinated mice than that in untreated mice (Fig. 3E, 3F), along with increased expression levels of the activation antigen CD69 on CD4^+^ T cells (Fig. 3G). Taken together, these results indicated that CpG M362, as a vaccine adjuvant, enhanced HBV-specific cellular responses.

### cHBV-vaccine alleviated immunosuppression and restored the exhausted HBV-specific CXCR5^+^ CD8^+^ T cells

**Figure 4:**
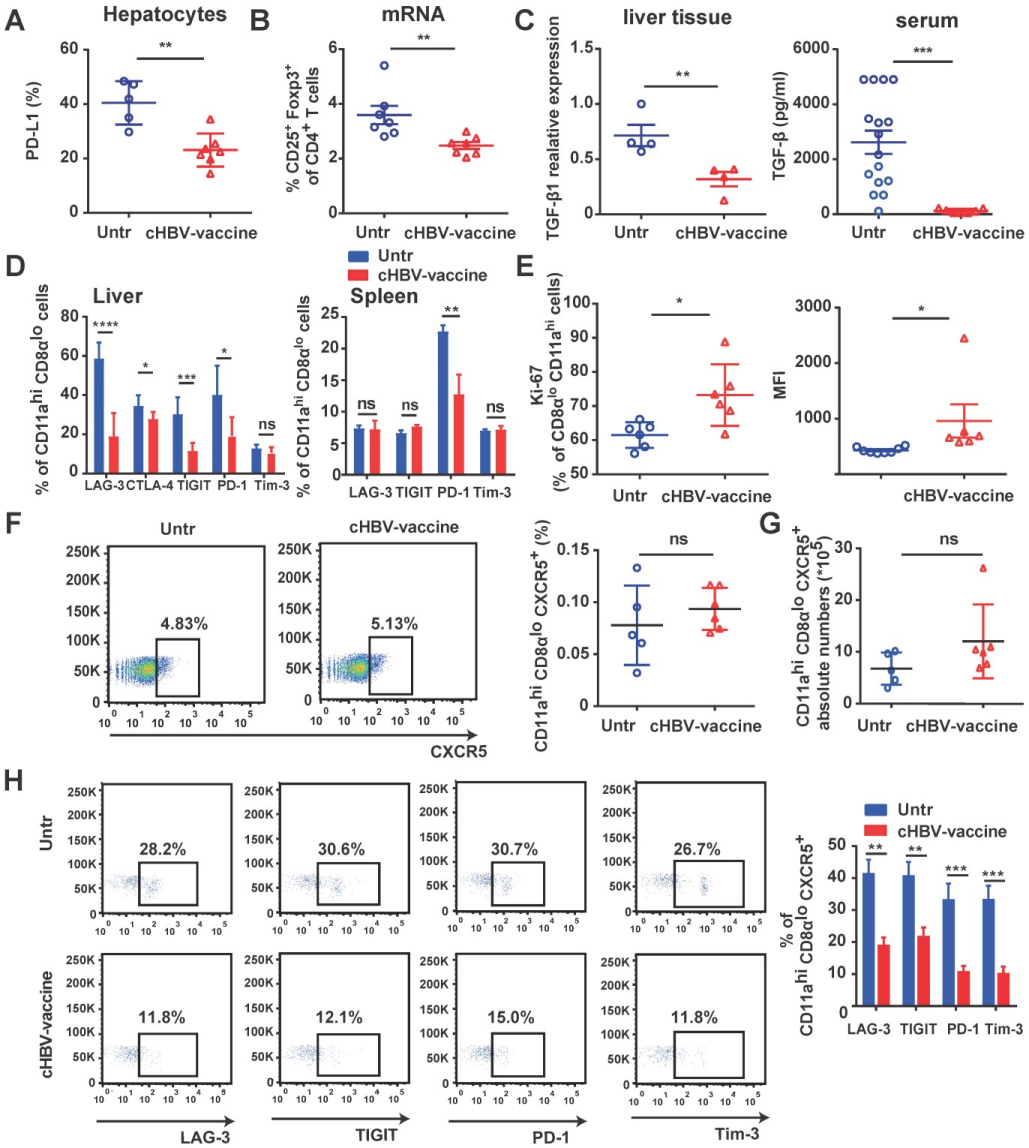
** cHBV-vaccine alleviated immunosuppression and restored exhausted HBV-specific CXCR5^+^ CD8^+^ T cells.** HBV-carrier mice were immunized subcutaneously with PBS (Untr) and 2 μg rHBVvac combined with 10 μg CpG M362 (cHBV-vaccine) weekly for 3 weeks, separately. (A) The expression of PD-L1 on hepatocytes on day 21 post-immunization. (B) The proportion of splenic CD4^+^ CD25^+^ Foxp3^+^ on day 21 post-immunization. (C) Relative expression of TGF-β1 mRNA in liver tissue and serum TGF-β1 levels on day 21 post-immunization. (D) LAG-3, CTLA-4, TIGIT, PD-1 and Tim-3 expression on HBV-specific CD11a^hi^ CD8α^lo^ cells in liver (left) and spleen (right) on day 21 post-immunization. (E) Ki-67 expression on HBV-specific CD11a^hi^ CD8α^lo^ cells in liver on day 21 post-immunization. (F-G) Representative flow-cytometry scatter plot of splenic CXCR5^+^ CD11a^hi^ CD8α^lo^ T cells. The frequency (F) and abundance (G) of CXCR5^+^ CD11a^hi^ CD8α^lo^ T cells on day 21 post-immunization. (H) Representative flow-cytometry scatter plot of PD-1, TIGIT, Tim-3, and LAG-3 expression on HBV-specific CXCR5^+^ CD11a^hi^ CD8α^lo^ T cells on day 21 post-immunization. All data are expressed as mean ± SEM (n ≥ 5). *p < 0.05, **p < 0.01, ***p < 0.001 versus untreated mice.

The immunosuppressive environment and exhaustion of CD8^+^ T cells in the HBV-infected liver are the major causes underlying the refractoriness of CHB 5, 39, 42. cHBV-vaccination downregulated the expression of PD-L1 on hepatocytes of HBV-carrier mice (Fig. 4A), reduced the proportion of Tregs (Fig. 4B), and decreased TGF-β1 levels in liver tissues and serum (Fig. 4C). Furthermore, HBV-specific CD11a^hi^ CD8α^lo^ T cells in cHBV-vaccinated mice displayed significantly reduced expression of LAG-3, CTLA-4, TIGIT, and PD-1 compared to that in untreated mice (Fig. 4D), accompanied by upregulation of pro-proliferative nuclear antigen Ki-67 (Fig. 4E). Follicular CXCR5^+^ CD8^+^ T cells have been recently found to play a pivotal role in controlling viral replication during chronic infections such as HIV, LCMV, and HBV 24, 27. Interestingly, approximately 5% of the HBV-specific CD11a^hi^ CD8α^lo^ cells in the spleens of HBV-carrier mice abundantly expressed CXCR5. However, the subset of CXCR5^+^ HBV-specific CD11a^hi^ CD8α^lo^ cells was more exhausted than the predominant CXCR5^-^ subset (Figure S3). Although the frequency and abundance of CXCR5^+^ CD11a^hi^ CD8α^lo^ cells were not significantly altered by cHBV vaccination (Fig. 4F, 4G), the expression of multiple co-inhibitory receptors such as LAG-3, PD-1, TIGIT, and TIM-3 was significantly downregulated (Fig. 4H). Meanwhile, cHBV vaccination did not significantly affect the frequency of CXCR5^+^ CD4^+^ T cells, but decreased the expression of TIGIT and PD-1 on CXCR5^+^ CD11a^hi^ CD4^+^ T cells (Fig. S4). These results indicated that CpG M362 as a vaccine adjuvant was able to overcome the mechanisms that impaired the functional immune responses in HBV-carrier mice.

### cHBV-vaccine induced long-term immune memory against HBV re-challenge

**Figure 5:**
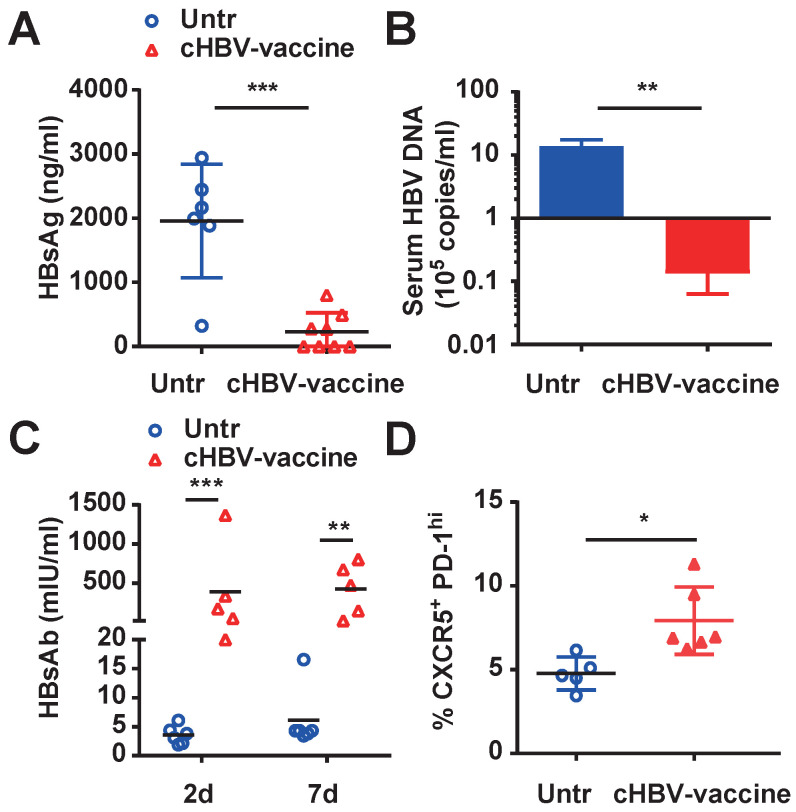
** cHBV-vaccine induced long-term immune memory against HBV re-challenge.** HBV-carrier mice were immunized subcutaneously with PBS (Untr) and 2 μg rHBVvac combined with 10 μg CpG M362 (cHBV-vaccine) weekly for 3 weeks, separately. Then, these treated mice were re-challenged with hydrodynamic injection of 8 μg pAAV/HBV1.2 plasmid on day 59 after the first vaccination. Serum levels of HBsAg (A) and HBV DNA copies (B) detected by CLIA and *q*-PCR respectively on day 7 after HBV re-challenge. (C) Serum HBsAb levels on day 7 after HBV re-challenge. (D) The frequency of CXCR5^+^ PD-1^+^ CD4^+^ T cells (T_fh_ cells) in spleen on day 7 after HBV re-challenge. All data are expressed as mean ± SEM (n ≥ 5). *p < 0.05, **p < 0.01, ***p < 0.001 versus untreated mice.

The major challenge of clinical HBV therapy is to prolong the immunological memory against the recurrence of HBV infection 43, 44. To this end, HBV-carrier mice were re-challenged with HBV on day 59 after cHBV vaccination. Both HBsAg and HBV DNA were nearly undetectable in the serum of cHBV-vaccinated mice (Fig. 5A, 5B) compared to that in untreated mice, accompanied by higher levels of protective anti-HBs (Fig. 5C). Furthermore, the proportion of CXCR5^+^ PD-1^+^ follicular helper T cells increased upon HBV re-challenge (Fig. 5D). Meanwhile, serum ALT remained at the baseline levels in cHBV-vaccinated mice after HBV re-challenge (Fig. S5). Taken together, the results suggested that CpG M362 as a vaccine adjuvant induced long-term immune memory against HBV re-infection.

### cHBV-vaccine induced long-lasting CXCR5^+^ CD8^+^ T cell response during HBV re-challenge

**Figure 6:**
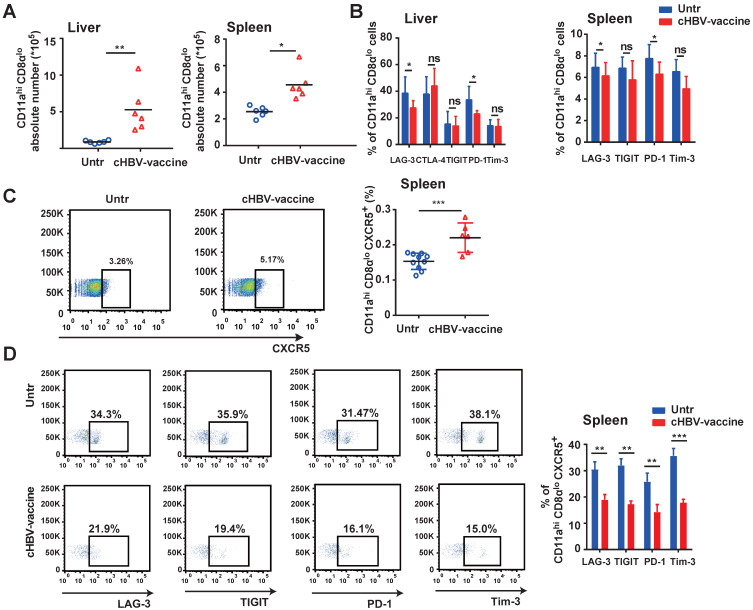
** cHBV-vaccine induced long-lasting CXCR5^+^ CD8^+^ T cell response during HBV re-challenge.** HBV-carrier mice were immunized subcutaneously with PBS (Untr) and 2 μg rHBVvac combined with 10 μg CpG M362 (cHBV-vaccine) weekly for 3 weeks, separately. Then, these treated mice were re-challenged with hydrodynamic injection of 8 μg pAAV/HBV1.2 plasmid on day 59 after the first vaccination. (A) Abundance of CD11a^hi^ CD8α^lo^ cells among CD8^+^ T cells from liver and spleen on day 7 after HBV re-challenge. (B) LAG-3, CTLA-4, TIGIT, PD-1 and Tim-3 expression on HBV-specific CD11a^hi^ CD8α^lo^ cells in liver (left) and spleen (right) on day 7 after HBV re-challenge. (C) Representative flow-cytometry scatter plot of splenic CXCR5^+^ CD11a^hi^ CD8α^lo^ T cells, showing the frequency of CXCR5^+^ CD11a^hi^ CD8α^lo^ T cells on day 7 after HBV re-challenge. (D) Representative flow-cytometry scatter plot of PD-1, TIGIT, Tim-3, and LAG-3 expression on HBV-specific CXCR5^+^ CD11a^hi^ CD8α^lo^ T cells on day 7 after HBV re-challenge. *p < 0.05, **p < 0.01, ***p < 0.001 versus untreated mice.

An effective vaccine should generate appreciable numbers of high-quality memory CD8^+^ T cells that can be immediately activated upon re-exposure to the pathogen 45-47. Compared to untreated mice, the abundance of HBV-specific CD11a^hi^ CD8α^lo^ cells in the liver and spleen of cHBV-vaccinated mice increased significantly after HBV re-challenge (Fig. 6A), accompanied by downregulated expression of PD-1 and LAG-3, indicating an effective immune response (Fig. 6B). Furthermore, cHBV vaccination significantly increased the frequency of splenic CXCR5^+^ CD11a^hi^ CD8α^lo^ cells (Fig. 6C) and decreased the expression of multiple co-inhibitory receptors (Fig. 6D). Although the frequency of splenic CXCR5^+^ CD11a^hi^ CD4^+^ T cells increased in response to HBV re-challenge, cHBV vaccination only decreased PD-1 levels on CXCR5^+^ CD11a^hi^ CD4^+^ T cells (Fig. S6). These data indicated that the adjuvant properties of CpG M362 induced a long-lasting antiviral CXCR5^+^ CD8^+^ T cell response against HBV.

## Discussion

CHB infection results from a dynamic balance between viral replication and the host immune response. Liver-induced systemic immune tolerance is the basis of CHB, which is characterized by high viral load and impaired HBV-specific adaptive T cell responses 5, 39. The ideal endpoints of HBV treatment are HBsAg loss, seroconversion to anti-HBs, and sustained inhibition of HBV DNA 48. Since HBV antigens are weakly immunogenic, adjuvants are needed to accelerate, prolong, and enhance the immune response against CHB. Among the TLR9 agonists tested as adjuvants in vaccines against cancer cells and intracellular pathogens, CpG ODNs induce activation of both the Th1-polarized immune response, including NK cells, macrophages, and APCs, and the humoral immune response to control local tumor growth 22, 49, 50. Studies have shown that CHB impairs the host innate immune system by downregulating TLR expression and inhibiting downstream signaling pathways 51, 52. Meanwhile, treatment with synthetic TLR9 agonists, such as class A and B CpG ODNs, reportedly enhanced anti-HBV immunity and HBV elimination during CHB therapy 10, 19, 21. Therefore, the current study investigated the effectiveness of CpG M362, a class C CpG ODN that combines the features of both class A and B CpG ODNs, as an adjuvant with rHBVvac in HBV-carrier mice.

DCs expressing TLRs play an important role in triggering adaptive immunity to pathogens 53. Recent studies have demonstrated that administering a TLR3 agonist enhanced the maturation of CD8α^+^ DCs, which in turn promoted cross-presentation in the tumor microenvironment and tumor regression 54,55. Concurring with these findings, cHBV vaccination upregulated the expression of CD40, CD80, and CD86 on mDCs and pDCs. Moreover, pDCs exhibited stronger activation than mDCs upon treatment with the cHBV-vaccine, mainly due to the presence of CpG M362. In addition, cHBV vaccination decreased PD-L1 expression on hepatocytes, which is pivotal to the progressive loss of HBV-specific T cell function during CHB infection and has been shown to affect treatment response in later clinical therapy 5, 56. Furthermore, mice administered the cHBV-vaccine exhibited significantly reduced Treg cell frequency and lower serum TGF-β1 levels compared to untreated mice. Meanwhile, both HBsAg and HBV DNA levels were nearly undetectable in the vaccinated mice during the long-term memory immune response assay on day 66, accompanied with higher levels of protective anti-HBs. Taken together, these results indicate that cHBV vaccination inhibits CHB-induced immune tolerance and triggers long-term anti-HBV-specific immunity in HBV-carrier mice. Furthermore, the results support that treatment with rHBVvac alone does not efficiently eliminate HBV, nor induce the same immunological effects as those induced by the cHBV-vaccine.

Effector CD8^+^ T cells predict the efficacy of therapeutic vaccines against pathogens. However, CHB infection inhibits HBV-specific T cell responses, resulting in exhausted phenotypes, poor cytotoxic activity, and impaired cytokine secretion, accompanied by the expression of co-inhibitory receptors such as PD-1, LAG-3, CTLA-4, TIGIT, and TIM-3 5, 39. Blockade of PD-1 reportedly improved the proliferation of CD8^+^ T cells and increased production of IFN-γ and IL-2, offering a novel therapeutic strategy for CHB 57-59. Interestingly, the cHBV-vaccine also downregulated the expression of LAG-3, CTLA-4, TIGIT, and PD-1 immune checkpoints on HBV-specific CD8^+^ T cells.

Antigen-experienced CD8^+^ T cells after infection or vaccination exhibit upregulated expression of CD11a and downregulated expression of CD8α on CD8^+^ T cells, whereas inflammatory stimulation alone, such as CpG administration, does not drive these changes 40. During CHB infection, HBV-specific CD8^+^ T cells are known to gradually lose effector functions, proliferative capacity, and cytolytic activity, in that order 5, 56. In the current study, cHBV vaccination increased the abundance of CD11a^hi^ CD8α^lo^ cells, as well as the proliferative capacity and activation of HBV-specific CD8^+^ T cells. Furthermore, cHBV-vaccinated mice exhibited significantly increased serum levels of IFN-γ without elevated ALT levels, which concurred with previous studies reporting that IFN-γ mediates non-cytolytic clearance of HBV from hepatocytes without liver damage 5, 60. Therefore, the therapeutic effects of the cHBV-vaccine are likely mediated via a non-cytolytic HBV CD8^+^ T cell effect.

Follicular CXCR5-expressing CD8^+^ T cells are a major reservoir of long-term immunity 24, 61. Compared to the CXCR5- subset, CXCR5^+^ CD8^+^ T cells are localized to the splenic B cell follicles and tend to display an exhausted phenotype, expressing intermediate levels of PD-1 and TIGIT. In addition, they have enhanced effector potential, self-renewal capacity, and are negatively correlated with HBV progression 27, 61. HBV-specific CD11a^hi^ CD8α^lo^ cells in the spleens of HBV-carrier mice abundantly expressed CXCR5, and were the predominant subset among exhausted cells. However, cHBV vaccination significantly reduced the expression of multiple co-inhibitory receptors on CXCR5^+^ CD11a^hi^ CD8α^lo^ cells, indicating that HBV was eliminated by restoring exhausted CXCR5^+^ HBV-specific CD8^+^ T cells. Effector CD4^+^ T cells also participate in humoral and cellular immune responses by generating and maintaining both neutralizing antibodies and CD8^+^ T cells to facilitate HBV clearance 62, 63. Moreover, antigen-specific CD4^+^ T cells reportedly display CD11a^hi^ CD49d^+^ surface marker expression 41. Thus, cHBV vaccination increased the generation and activation of HBV-specific CD4^+^ T cells, indicating that CD4^+^ T cells might contribute to the humoral and CD8^+^ T cell response. Combined, these results suggest that augmented HBV-specific CD8^+^ and CD4^+^ T-cell responses induced by the cHBV-vaccine contribute to the elimination of HBV. Moreover, effective vaccines against viruses should generate a stable high-quality population of memory CD8^+^ T cells to confer long-term protective immunity 43, 46, 47, 64. The abundance of HBV-specific CD11a^hi^ CD8α^lo^ cells, which were predominantly CXCR5^+^ CD11a^hi^ CD8α^lo^ T cells, increased remarkably after HBV re-challenge in cHBV-vaccinated mice, suggesting the cHBV-vaccine promoted a long-term memory response against the virus in HBV-carrier mice, accompanied by higher levels of protective anti-HBs.

In conclusion, CpG ODNs have been widely used as adjuvants for cancer and antiviral vaccines, such as CpG 1018 (CpG-B ODN) in HEPLISAV-B 14-18. Moreover, a dose of spike (S)-protein (S-Trimer) combined with CpG 1018/Alum adjuvants induced robust humoral and cellular immune responses against SARS-CoV-2 in a phase II/III trial 65, 66. In the current study, the CpG M362-based HBV vaccine overcame systemic immune tolerance in HBV-carrier mice and restored exhausted HBV-specific CD8^+^ T cells, which were predominantly CXCR5^+^ CD11a^hi^ CD8α^lo^ T cells. More importantly, this approach effectively induced long-term immune memory against HBV recurrence. Considering that CpG M362 combines the features of both class A and B CpG ODNs, the cHBV-vaccine provides a promising candidate for anti-HBV immunotherapy and the prevention of HBV.

## Supplementary Material

Supplementary figures.

## Abbreviations

anti-HBs: hepatitis B surface antibody
APCs: antigen-presenting cells
BMDCs: bone marrow-derived dendritic cells
CHB: chronic hepatitis B virus
CpG: cysteine-guanine dinucleotide
CTLA-4: cytotoxic T-lymphocyte-associated antigen-4
DCs: dendritic cells
dLNs: draining lymph nodes
HBV: hepatitis B virus
HBsAg: hepatitis B surface antigen
HCV: hepatitis C virus
HIV: human immunodeficiency virus
IFN-γ: interferon-γ
IL-12: interleukin-12
mAB: monoclonal antibody
mDCs: myeloid dendritic cells
MNCs: mononuclear cells
ODN: oligodeoxynucleotide
PD-1: programmed cell death-1
pDCs: plasmacytoid dendritic cells
PD-L1: programmed death-ligand 1
RBC: red blood cell
Th1: T helper 1 cell
Tfh: follicular helper T cell
TIM-3: T-cell immunoglobulin domain and mucin domain 3
TNF-α: tumor necrosis factor-α
Treg: regulatory T cell
