# Supplementary material for: CpG-C ODN M362 as an immunoadjuvant for HBV therapeutic vaccine reverses the systemic tolerance against HBV
Source: Int J Biol Sci. 2022 Jan 1;18(1):154–65. doi: 10.7150/ijbs.62424 (PMC8692134; doi:10.7150/ijbs.62424)
Supplement: Supplementary file 1 — Supplementary figures. [file ijbsv18p0154s1.pdf]

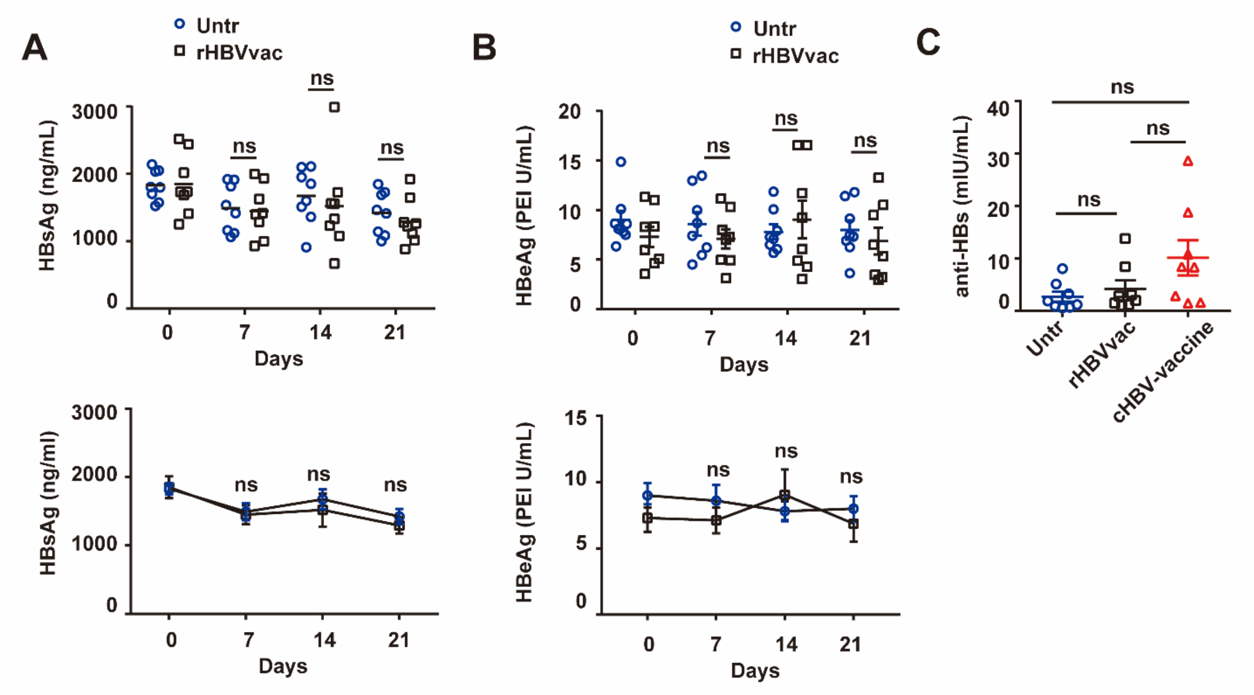

Figure S1. rHBVvac alone did not eliminate HBV in HBV-carrier mice. HBV-carrier mice were immunized subcutaneously with PBS (Untr), 2 μg rHBV vaccine (rHBVvac), and 2 μg rHBVvac combined with 10 μg CpG M362 (cHBV-vaccine) weekly for 3 weeks, separately. (A) Post-immunization serum levels of HBsAg, measured by ELISA and compared with two-way ANOVA. (B) Post-immunization serum levels of HBeAg, measured by ELISA and compared with two-way ANOVA. (C) Serum anti-HBs levels on day 21 after the treatment. All data represent a mean ± SEM (n ≥ 8). *$p < 0.05$ versus Untr mice.

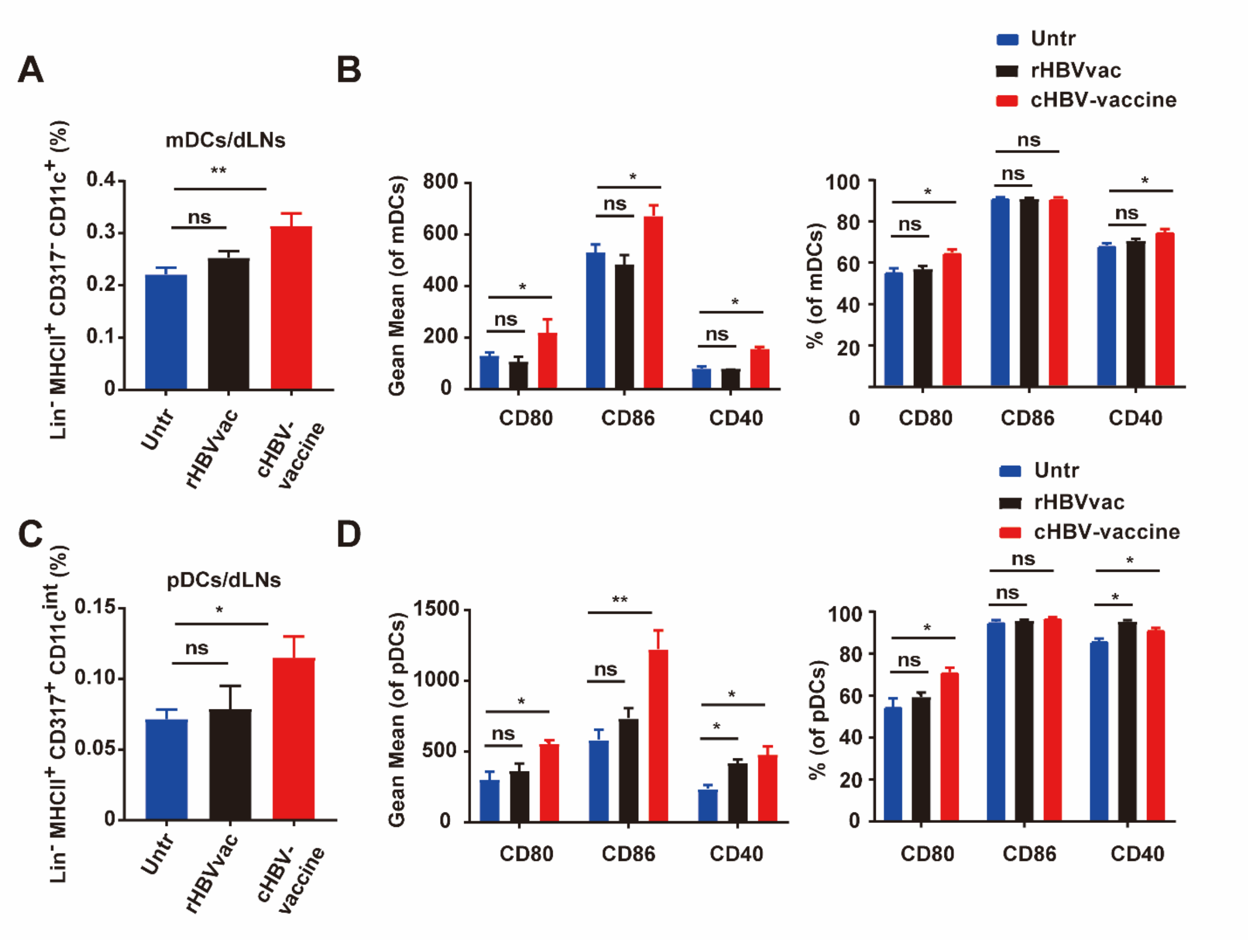

Figure S2. CpG M362 promoted the activation of DCs in dLNs. HBV-carrier mice were immunized subcutaneously with PBS (Untr), 2 μg rHBV vaccine (rHBVvac), and 2 μg rHBVvac combined with 10 μg CpG M362 (cHBV-vaccine) weekly for 3 weeks, separately. (A) Proportion of mDCs (Lin$^{-/-}$ MHCII^{+} CD317^{-} CD11c^{+}) on day 21 post-immunization in dLNs. (B) Flow cytometry results showing expression of CD80, CD86 and CD40 on mDCs in dLNs. (C) Proportion of pDCs (Lin$^{-/-}$ MHCII^{+} CD317^{+} CD11c^{int}) on day 21 post-immunization in dLNs. (D) Flow cytometry results showing expression of CD80, CD86 and CD40 on pDCs in dLNs. All data represent a mean ± SEM (n ≥ 8). *$p < 0.05$, **$p < 0.01$ versus Untr mice.

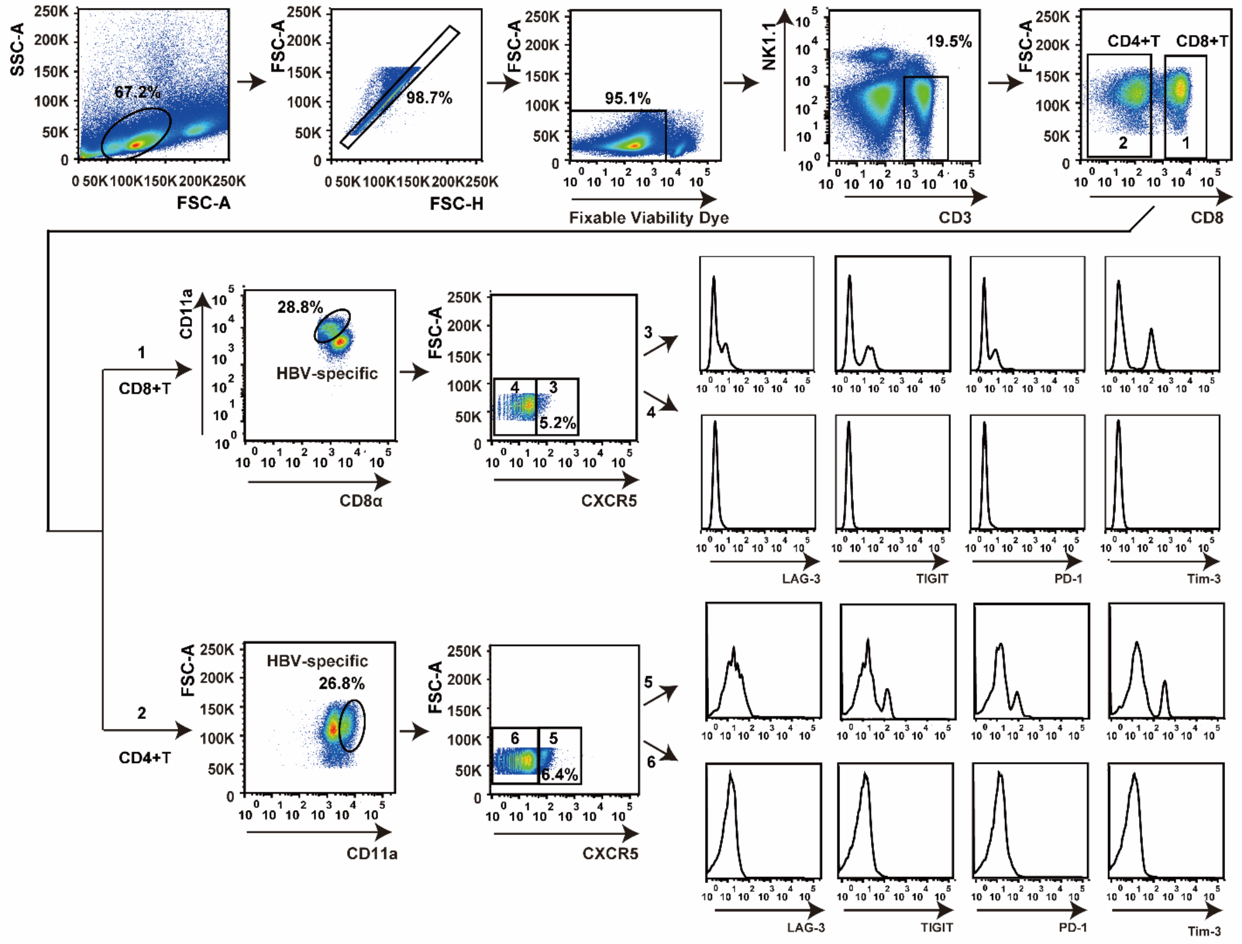

Figure S3. HBV-specific CXCR5$^+$ CD11a^{hi} CD8α^{lo} T cells and CXCR5$^+$ CD11a^{hi} CD4$^+$ T cells expressed higher levels of inhibitory molecules LAG-3, TIGIT, PD-1 and Tim-3 than their CXCR5$^-$ counterpart during CHB infection. Splenocytes were harvested from HBV-carrier mice on day 21 post-immunization. Cell doublets were gated out using FSC-A vs FSC-H followed by using the Fixable Viability Dyes to exclude the dead cells, and then CD3$^+$ CD8$^+$ T lymphocytes were gated from these cells. HBV-specific CD11a^{hi} CD8α^{lo} T cells and HBV-specific CD11a^{hi} CD8$^-$ T cells (clarified as CD4$^+$ T cells) were gated from these cells, then these cells were further separated into CXCR5$^+$ cells and CXCR5$^-$ cells, and the expression of PD-1, TIGIT, Tim-3, and LAG-3 on these two cells were analyzed by flow cytometry respectively.

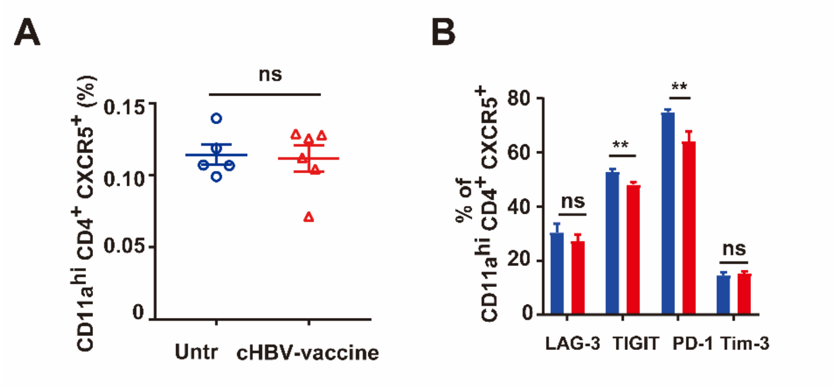

Figure S4. cHBV-vaccine partly restored the exhausted HBV-specific CXCR5^{+} CD4^{+} T cells. HBV-carrier mice were immunized subcutaneously with PBS (Untr) and 2 μg rHBVvac combined with 10 μg CpG M362 (cHBV-vaccine) weekly for 3 weeks, separately. (A) The frequency of splenic CXCR5^{+} CD11a^{hi} CD4^{+} T cells on day 21 post-immunization. (B) PD-1, TIGIT, Tim-3 and LAG-3 expression on HBV-specific CXCR5^{+} CD11a^{hi} CD4^{+} T cells on day 21 post-immunization. All data are expressed as mean ± SEM (n ≥ 5). $^{**}p < 0.01$ versus unvaccinated mice.

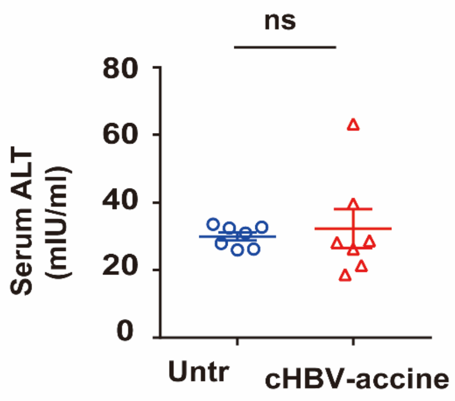

Figure S5. The serum ALT remained at the baseline levels in cHBV-vaccine-treated group after HBV re-challenge. HBV-carrier mice were immunized subcutaneously with PBS (Untr) and 2 μg rHBVvac combined with 10 μg CpG M362 (cHBV-vaccine) weekly for 3 weeks, separately. Then, these treated mice were re-challenged with hydrodynamic injection of 8 μg pAAV/HBV1.2 plasmid on day 59 after the first vaccination. Serum levels of ALT monitored on day 7 after the HBV re-challenge (Normal serum ALT levels are < 40 mIU/mL). All data represent a mean ± SEM (n ≥ 7). *$p < 0.05$ versus Untr mice.

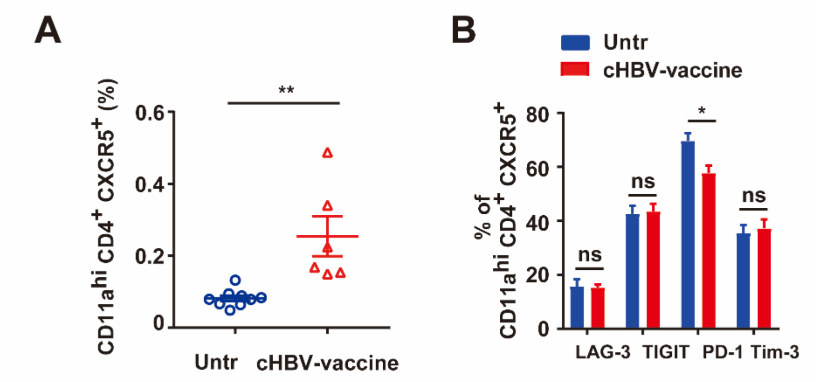

Figure S6. cHBV-vaccine induced long-lasting CXCR5$^+$ CD4$^+$ T cell response during HBV re-challenge. HBV-carrier mice were immunized subcutaneously with PBS (Untr) and 2 μg rHBVvac combined with 10 μg CpG M362 (cHBV-vaccine) weekly for 3 weeks, separately. Then, these treated mice were re-challenged with hydrodynamic injection of 8 μg pAAV/HBV1.2 plasmid on day 59 after the first vaccination. (A) The frequency of splenic CXCR5$^+$ CD11a^{hi} CD4$^+$ T cells on day 7 after HBV re-challenge. (B) PD-1, TIGIT, Tim-3, and LAG-3 expression on HBV-specific CXCR5$^+$ CD11a^{hi} CD4$^+$ T cells on day 7 after HBV re-challenge. All data are expressed as mean ± SEM (n ≥ 6). *$p < 0.05$, **$p < 0.01$ versus unvaccinated mice.
